# The Impact and Applications of Phages in the Food Industry and Agriculture

**DOI:** 10.3390/v12020210

**Published:** 2020-02-13

**Authors:** Jennifer Mahony, Eoghan Casey, Douwe van Sinderen

**Affiliations:** School of Microbiolgy and APC Microbiome Ireland, University College Cork, Western Road, T12 YT20 Cork, Ireland; Eoghan.casey@ucc.ie

**Keywords:** dairy, fermentation, lactic acid bacteria, food pathogens, food spoilage, bacteriophage

Food security is currently a global socio-political pressure point that is exacerbated by the ever-increasing world population. The demand for increased food production levels, in parallel with consumer demands for minimally processed foods and natural preservation methods, requires the food industry to identify novel approaches to ensuring food safety and quality. The application of bacteriophages (or phages) and phage-derived proteins, such as biosanitisers, is a natural alternative or enhancement to current food preservation approaches and there is growing evidence and, indeed, application of phages/phage cocktails/phage proteins in the food preservation context. Such approaches, and their intended outcomes, align well with the United Nations Envision 2030 program’s sustainability development goals (SDGs), goal 12 in particular, under which it is proposed that, by 2030, global food waste at the retail and consumer levels should be halved and food losses, including post-harvest losses, should be dramatically reduced. Commercially available products, including Phage Guard S, E and Listex^TM^, which are aimed at reducing or eliminating *Salmonella*, *Escherichia coli* 0157 and *Listeria monocytogenes*, respectively, in food products have paved the way for the development of additional phage-based products. Therefore, it is imperative that we continue to increase our efforts to isolate phages that may be applied in this context and attempt to understand their genomic diversity, their interactions and explore their potential applications.

In this Special Issue, the potential applications of phages or phage-derived lytic enzymes for the control of spoilage microorganisms, such as *Dickeya solani* [1], and pathogenic organisms, including *Listeria monocytogenes* [2,3], *Streptococcus suis* [4] and *Yersinia* [5], are described. Indeed, combination therapies are also endorsed, wherein the combined application of *Listeria*-specific phages and nisaplin present further evidence that we may be able to enhance currently employed food protection measures [2]. Furthermore, studies detailing the isolation and diversity of phages capable of infecting spoilage organisms, including *Lactobacillus brevis* [6], highlight the potential that exists among the phages of currently under-studied species. To effectively exploit phages, it is essential to understand the interactions of phages and their hosts and considerable research efforts are ongoing in this area. Stone and colleagues outlined state-of-the-art phage-host interactions and the role of such studies in shaping phage-based treatments [7]. While phages may have far-reaching implications for food preservation and safety, public concerns regarding phage dissemination has, in many ways, retarded or even completely obstructed the pace of development in this area. Here, Sommer et al. highlight the current strategies used in the food industry to inactivate phages, the effectiveness of these approaches and proposed improvements to current approaches to mitigate consumer and regulatory body concerns [8].

While phages are regarded as potential saviors in the fight against food-borne pathogens and spoilage microorganisms, their presence in food fermentation, particularly dairy fermentation, is undesirable. Infection of starter cultures in food fermentations may have a negative impact on the organoleptic properties of the final product, including the flavor, aroma and/or texture. Modern consumers are highly discerning and inconsistent products can have a considerable economic impact on producers. In more serious cases, phage infection of starter cultures may lead to partial or complete fermentation failure, leading to increased waste production. Consequently, the interactions of phages of organisms such as *Lactococcus lactis* and *Streptococcus thermophilus* have received significant research attention in recent decades. This has culminated in the development of data-driven solutions to reducing the risk of phage infection in fermentation plants. In this Special Issue, the effectiveness of thermal and chemical treatments that are relevant to the food industry are explored by Marcό and colleagues [9], while the PCR-based detection and diversity analysis of lactococcal 936-group phages highlights emerging tools that may be applied in the dairy industry to reduce the risk of phage infection [10]. Furthermore, the diversity of phages infecting *Lactobacillus plantarum*, as detailed by Krykou et al., highlights the knowledge gaps in phage diversity in important bacterial species and the potential application of such phages in producing robust strains for application in the food and probiotics industries [11]. Temperate phages are often regarded negatively in a food production context; however, the role of temperate phages in providing resistance against homologous, and even heterologous phages, must also be considered. In this Special Issue, the Corich research group detail the role of a cryptic prophage in conferring phage resistance and highlight the importance of such features in defining and developing robust starter cultures [12].

In summation, phages are considered the foe of food fermentations, while they present considerable benefits to the safety of minimally processed vegetable and meat products. In both scenarios, it is essential that we understand the diversity and interactions of these fascinating biological entities so that we can continue to meet consumer demands, reduce food waste and improve the safety and integrity of our foods. As phages are a natural part of every ecological niche, we cannot ignore their role and impact on the food production chain.
